# From COVID-19 to Health System Reform: Qualitative Insights on Resilience and Lessons Learned from Finland

**DOI:** 10.1093/eurpub/ckac129.350

**Published:** 2022-10-25

**Authors:** L-K Tynkkynen, L Kihlström, I Keskimäki

**Affiliations:** 1 Faculty of Social Sciences, Tampere University, Tampere, Finland; 2 Welfare State Research and Reform, Finnish Institute for Health and Welfare, Helsinki, Finland

## Abstract

The pressure of COVID-19 on health system functioning has made policies to strengthen health system resilience a major theme of research. Accordingly, crises like COVID-19 can be seen as windows of opportunity for health system reforms to enhance health system resilience. In Finland, COVID-19 arrived on the eve of a major health system reform. In 2023 running health and social services will be transferred from 309 municipalities to 22 counties. While the reform was framed before the pandemic, we explore how lessons from COVID-19 matter for the future reform. Our results come from interviews of 53 top managers and civil servants in the year one of COVID-19, representing municipalities, municipal healthcare authorities and state agencies. The results offer a lookout to how national and local healthcare leaders view pandemic responses in connection to the future reform. Finland fared well in the pandemic compared to many countries e.g. in terms of excess deaths. However, our results reveal a tension between major issues in managing COVID-19 and implementing the reform. While the data suggest that dealing with a prolonged crisis proved challenging due to lack of trust, communication, and transparency between national and subnational actors in the health system, the dominant lessons learned and needs for reform among the interviewees build upon obvious fixes, such as ensuring supply of PPEs and ICU beds for the next pandemic. While being important in preparedness, these can build a legacy not tackling the root causes of lacking resilience and can be inconsistent with reform goals. The pandemic provides an opportunity to analyse the reformed system from a new viewpoint and may reveal weaknesses not considered in reform planning. Reforms can impact health system resilience in positive and negative ways. While different shocks may open new avenues for system transformation, they can also create path dependencies weakening the systems’ ability to prepare for unknown threats.

